# Comparison of Three Different Staging Systems Capable of Predicting the Severity of Congenital Lower Urinary Tract Obstruction (LUTO) and Its Prognosis

**DOI:** 10.1002/jcu.70045

**Published:** 2025-08-26

**Authors:** Ugo Maria Pierucci, Irene Paraboschi, Matthieu Peycelon, Chryso P. Katsoufis, Alireza Alam, Gabriele Tonni, Mark D. Kilby, Gloria Pelizzo, Rodrigo Ruano

**Affiliations:** ^1^ Department of Pediatric Surgery Buzzi Children's Hospital Milan Italy; ^2^ Department of Biomedical and Clinical Sciences University of Milano Milan Italy; ^3^ Department of Pediatric Surgery and Urology National Reference Center for Rare Urinary Tract Malformations (CRMR MARVU), ERN eUROGEN Accredited Center, Robert‐Debré University Hospital, APHP, GHU North, UMR INSERM 1141 NEURODEV, Université Paris Cité Paris France; ^4^ Division of Pediatric Nephrology University of Miami Miller School of Medicine Miami Florida USA; ^5^ Division of Pediatric Urology Jackson Memorial Hospital Miami Florida USA; ^6^ Prenatal Diagnostic Centre, Department of Obstetrics and Neonatology Istituto di Ricovero e Cura a Carattere Scientifico (IRCCS) Reggio Emilia Italy; ^7^ Birmingham Women's & Children's Foundation Trust and University of Birmingham Birmingham UK; ^8^ Division of Maternal‐Fetal Medicine, Department of Maternal and Fetal Medicine, Obstetrics and Gynecology University of Miami, Miller School of Medicine Miami Florida USA

**Keywords:** fetal intervention, fetal renal function, fetal ultrasound, lower urinary tract obstruction (LUTO), prenatal diagnosis, prognostic staging systems

## Abstract

Lower urinary tract obstruction (LUTO) is a rare but severe fetal condition associated with significant morbidity and long‐term renal risk. Several prenatal staging systems have been proposed to guide management and predict outcomes, yet their comparative prognostic value remains uncertain. This review provides a structured evaluation of three staging systems currently used in clinical practice: those proposed by Ruano, Fontanella, and Nassr. Ruano's system integrates detailed ultrasound findings and biochemical markers, providing explicit stage‐specific management recommendations and guidance for specific prenatal therapy. Fontanella's approach depends solely on ultrasound parameters, specifically bladder volume and the gestational timing of oligohydramnios onset, without incorporating biochemical markers or offering clear treatment guidelines. Nassr's score employs both ultrasound and biochemical data within a numerical scoring framework, focusing primarily on identifying severe cases that might benefit from prenatal intervention. Each system has distinct strengths and limitations. Further multicenter validation studies are necessary to determine which staging system most effectively predicts clinical outcomes and optimally guides management for fetuses diagnosed with LUTO.

## Graphical abstract

The Ruano's proposed prenatal staging system for LUTO seems to offer a more comprehensive stratification of different severities of fetal LUTO as well as a guidance for potential prenatal therapy based on four stages.

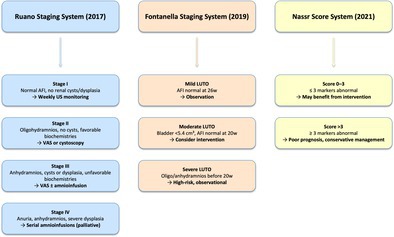

## 1 Introduction

Lower Urinary Tract Obstruction (LUTO) is a severe congenital anomaly associated with significant fetal morbidity and mortality (Haeri 2015; Pierucci et al. 2024). It represents a diagnostic and therapeutic challenge, necessitating accurate and comprehensive staging systems to guide clinical management and predict outcomes (Farrugia 2020, 2016; Farrugia and Kilby 2021; Capone et al. 2022). Prenatal counseling remains particularly demanding despite advancements in ultrasound detection and fetal intervention techniques (Tonni et al. 2013). Accurate prenatal staging of LUTO is inherently challenging due to considerable phenotypic heterogeneity, variability in timing of onset, and the presence of overlapping obstructive uropathies (Capone et al. 2022; Enninga and Ruano 2018). Furthermore, the correlation between prenatal findings and long‐term postnatal renal outcomes remains inconsistent, complicating prognostication and management planning (Enninga and Ruano 2018). Among available prenatal interventions, vesicoamniotic shunting (VAS) or fetal cystoscopy (FC) has been shown to improve perinatal survival in selected cases of LUTO compared to conservative management (Pierucci et al. 2025). However, the long‐term outcomes, including survival and renal function, remain uncertain (Nassr et al. 2017). This opinion paper compares the staging system proposed by (Ruano et al. 2017), (Fontanella et al. 2019), and the most recent score published by (Nassr et al. 2021), aiming to highlight their respective strengths and limitations and determine the most effective approach for clinical application. It evaluates these different staging systems, considering how each has contributed to advancing clinical practice and improving patient outcomes. The three systems included in this review were selected as they represent the only published prenatal staging classifications specifically developed for congenital LUTO. These systems were identified based on their structured design, clinical implementation, and relevance as reflected by citation within the maternal‐fetal medicine and fetal urology literature. No other staging systems fulfilling these criteria were found in the current peer‐reviewed literature. Given the absence of large‐scale, multicenter validation trials, this opinion paper aims to provide a structured comparison of the available prenatal staging systems, with emphasis on their conceptual underpinnings, clinical applicability, and prognostic implications. We have included in this review illustrative clinical examples cases, which demonstrate how each staging system can be applied in practice and how they may correlate with postnatal outcomes, depending on whether prenatal intervention was performed.

## 2 Overview of the Proposed Staging Systems and Scores

The staging system developed by (Ruano et al. 2017) offers a comprehensive approach to LUTO, covering all stages from mild to severe (Table 1). This system integrates detailed ultrasound findings and biochemical markers, thoroughly assessing the fetal condition and guiding treatment decisions (Ruano et al. 2017). Ultrasound parameters—such as kidney echogenicity, the presence of cortical cysts, and the amniotic fluid index—are commonly used to characterize the severity of LUTO (Ruano et al. 2017). In addition, fetal urinary biochemical markers (e.g., sodium, chloride, calcium, osmolality, and β2‐microglobulin) have been proposed as supplementary tools to evaluate renal function and the potential impact of the obstruction (Ruano et al. 2017). The treatment guidance is stage‐specific, making it highly practical for clinical use. In detail, Ruano's staging system, as in its last version published in 2017, includes (i) Stage I (mild LUTO) involves a normal amniotic fluid index after 18 weeks, normal kidney echogenicity, and no evidence of renal dysplasia, with prenatal surveillance recommended; (ii) Stage II (moderate LUTO) is characterized by oligohydramnios/anhydramnios, hyperechogenic kidneys without cortical cysts, and favorable fetal urinary biochemistry. The primary objective is to prevent severe pulmonary hypoplasia and attempt to delay the need for postnatal kidney replacement therapy, with fetal vesicoamniotic shunting (VAS) or fetal cystoscopy indicated; (iii) stage III (severe LUTO) includes severe renal dysplasia with hyperechogenic kidneys and unfavorable fetal urinary biochemistry. The goal is to prevent severe pulmonary hypoplasia, where a fetal vesicoamniotic shunt may be used; (iv) Stage IV (intrauterine fetal renal failure) is characterized by anhydramnios and severe renal dysplasia on ultrasound, with amnioinfusion recommended due to the poor prognosis. Therefore, with these severe fetal phenotypes, the prognosis and management plan can be more clearly outlined, with address to either palliative care (Shaw et al. 2024) or termination of pregnancy.

**TABLE 1:** Ruano staging system (Ruano et al. 2017).

Stage	Fetal Ultrasound	Fetal Biochemistries at 18–30 weeks	Possible fetal therapies
Stage I	Normal AFI. No renal cysts or dysplasia, no hyperechogenic kidneys	Favorable after sequential sampling	Weekly US monitoring
Stage II	Oligohydramnios, severe bilateral hydronephrosis. Absent cysts or dysplasia. Hyperechogenic kidneys may be present.	Favorable after maximum of 3 sequential samplings	Cystoscopy or VAS
Stage III	Anhydramnios or oligohydramnios. Hyperechogenic kidneys, some renal cysts	Unfavorable after sequential sampling	VAS with possible amnioinfusion
Stage IV	Anhydramnios and anuria after monitoring bladder refilling rate. Renal dysplasia.	Unfavorable biochemistries and documented anuria after monitoring refilling	Amnioinfusion

Abbreviations: AFI, amniotic fluid index; VAS, vesicoamniotic shunt.

The staging system proposed by Fontanella et al. (Fontanella et al. 2019) in 2019 is primarily based on ultrasound findings and aims to predict the likelihood of spontaneous resolution in cases of LUTO (Table 2). Unlike other classifications, it does not incorporate biochemical markers and relies solely on gestational age at the first detection of oligohydramnios and bladder volume at diagnosis. The system classifies cases into three stages: Stage I (mild LUTO) is defined by normal amniotic fluid volume at 26 weeks' gestation and a low risk of perinatal mortality; Stage II (moderate LUTO) is characterized by a bladder volume of less than 5.4 cm^3^ with normal amniotic fluid at 20 weeks, where prognosis is more variable and intervention may be considered; and Stage III (severe LUTO) includes cases with a bladder volume ≥ 5.4 cm^3^ and/or oligohydramnios before 20 weeks, carrying the highest risk of perinatal mortality and postnatal renal impairment.

**TABLE 2:** Fontanella staging system (Fontanella et al. 2019).

Staging	Definition
Severe LUTO	Bladder volume ≥ 5.4 cm^3^ and/or oligo‐anhydramnios before 20 weeks
Moderate LUTO	Bladder volume < 5.4 cm^3^ and/or Normal AF at 20 weeks
Mild LUTO	Normal AF at 26 weeks

*Note*: Staging of LUTO based on bladder volume at referral and GA at the first appearance of oligo‐ or anhydramnios. Oligohydramnios was defined by an amniotic fluid index (AFI) lower than 5 cm or a maximum vertical pocket (MVP) lower than 2 cm.

Abbreviations: AFI Amniotic fluid index; GA, Gestational age; LUTO, lower urinary tract obstruction; MVP, Maximum vertical pocket.

(Nassr et al. 2021) developed a score designed to identify candidates for fetal intervention (Table 3). This system combines prenatal ultrasound parameters and biochemical markers into a scoring system that predicts postnatal survival and guides treatment decisions. The ultrasound parameters include bladder volume, renal echogenicity, and bladder refill. Like those in Ruano's staging system, the biochemical markers include calcium, sodium, chloride, osmolality, and β2‐microglobulin. Scores above three indicate a poor prognosis, suggesting intervention is unlikely to be beneficial. In contrast, scores between 0 and 3 suggest potential benefit from fetal intervention, as these fetuses are more likely to survive postnatally.

**TABLE 3:** Nassr score (Nassr et al. 2021).

Scoring item	Details	Score
Fetal urine biochemistry	< 3 tested markers are abnormal	0
	≥ 3 tested markers are abnormal	2
Ultrasound morphology	No renal cortical cysts	0
	Renal cortical cysts present	4
Initial bladder volume	≥ 40 cc	0
	< 40 cc	1
Percent bladder refill	≥ 80% of the initial volume	0
	< 80% of the initial volume	1
Final score		0–8

*Note*: Details of the scoring system for determining the candidacy for fetal intervention. Fetal urine biochemistry markers and their normal cut‐offs are: Calcium (< 8 mg/L), Sodium (< 100 mmol/L), Chloride (< 90 mmol/L), Osmolarity (< 200 mOsm/L), β2 microglobulin (< 6 mg/L). Initial bladder volume is measured by 3D ultrasound. Cut‐offs for initial bladder volume (40 cc) and percent bladder refill (80%) were calculated using ROC analysis.

Abbreviations: 3D, Three‐dimensional ultrasound; LUTO, Lower urinary tract obstruction; ROC, Receiver operating characteristic analysis.

## 3 Comparative Analysis and Discussion

LUTO presents a complex challenge in prenatal diagnosis and management, requiring accurate and effective staging systems to guide clinical decisions and treatment. Various staging systems have been developed to address these needs, each with distinct strengths and limitations. While all three major systems—Ruano staging system (Ruano et al. 2017), Fontanella staging system (Fontanella et al. 2019), and Nassr's score (Nassr et al. 2021)—aim to assess LUTO severity and inform management, their ability to predict long‐term pulmonary and renal outcomes varies, and further validation is necessary.

A key factor when comparing these systems is their sensitivity and specificity in predicting pulmonary hypoplasia and significant renal impairment in childhood. Pulmonary hypoplasia is strongly linked to the degree and duration of oligohydramnios, while renal impairment hinges on fetal urine biochemistry and the presence of renal dysplasia. Recognizing these factors helps clarify how effectively each staging system can guide both prenatal and postnatal outcomes.

Ruano staging system (Ruano et al. 2017) stands out for its comprehensive approach, incorporating both ultrasound findings (e.g., amniotic fluid index, assessment for renal dysplasia) and fetal urinary biomarkers to offer a more precise risk stratification. This integrated evaluation supports more accurate, individualized treatment decisions, including stage‐specific recommendations such as vesicoamniotic shunting or fetal cystoscopy in select cases. Although the inclusion of fetal urine biochemistry has been correlated with postnatal renal function, the sensitivity and specificity of Ruano's system in predicting long‐term outcomes have yet to be validated in large, multicenter clinical studies. Nevertheless, its detail‐oriented framework—with Stage III and IV correlating strongly to poor renal and pulmonary outcomes—positions Ruano's system as the most thorough tool currently available.

In contrast, the Fontanella staging system (Fontanella et al. 2019) offers a more observational approach, emphasizing the prediction of spontaneous resolution based on ultrasound parameters such as bladder volume, gestational age, and oligohydramnios. This can be beneficial for identifying cases where LUTO may resolve without intervention, thus avoiding unnecessary procedures and reducing associated risks. However, the absence of biochemical markers limits its capacity to comprehensively evaluate renal function and overall disease severity. This gap may result in less informed decision‐making in instances where biochemical abnormalities are present but not evident on ultrasound. Furthermore, Fontanella's staging system does not propose a management protocol tailored to each fetal stage, potentially limiting its utility in severe LUTO cases requiring timely intervention.

The Nassr score (Nassr et al. 2021) focuses on severe fetal LUTO associated with severe oligohydramnios or anhydramnios, thereby effectively identifying candidates for antenatal intervention. This is particularly valuable when immediate decisions—as well as vesicoamniotic shunting—are needed. However, by concentrating on severe presentations, the Nassr score may overlook nuances crucial for the long‐term management of non‐severe yet clinically relevant LUTO cases. Its sensitivity is high for recognizing fetuses at risk of pulmonary hypoplasia, but the absence of longitudinal validation studies makes it difficult to determine its predictive accuracy for renal function into childhood.

**FIGURE 1:**
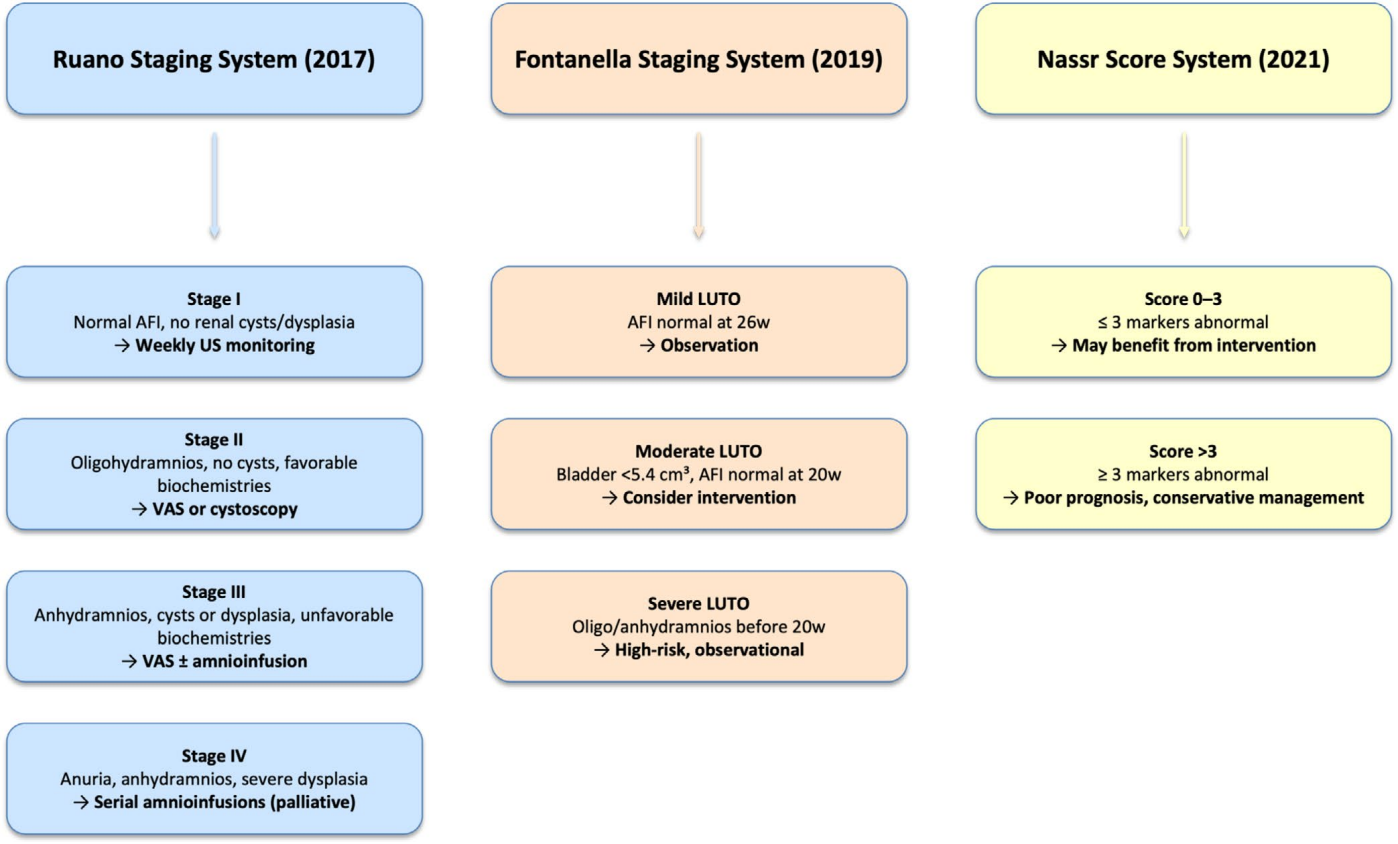
Comparative flowchart of three prenatal staging systems for fetal lower urinary tract obstruction (LUTO). Abbreviations: AFI, Amniotic fluid index; US, Ultrasound; VAS, Vesicoamniotic shunt.

A direct stage‐to‐stage comparison suggests that while Ruano's Stage III and IV classifications correlate strongly with poor renal and pulmonary outcomes, Fontanella's Stage III and Nassr's high‐risk scores capture severe disease but do not incorporate stage‐specific biochemical risk stratification. Among the currently available frameworks, Ruano's staging system integrates the broadest range of imaging and biochemical parameters, though its prognostic accuracy remains to be validated in prospective cohorts. Accordingly, the comparative value of each system remains interpretative. (Table 4, Figure 1).

**TABLE 4:** Summary table comparing the three different staging systems capable of predicting the severity of congenital lower urinary tract obstruction (LUTO) and its prognosis.

Criteria	Ruano staging system (Ruano et al. 2017)	Fontanella staging system (Fontanella et al. 2019)	Nassr score (Nassr et al. 2021)
Scope	Comprehensive, all stages	Focus on ultrasound findings	Focus on intervention candidates
Ultrasound parameters	Kidney echogenicity (normal to hyperechogenic), cortical cysts (present/absent), amniotic fluid index (normal to anhydramnios) after 18 weeks	Bladder volume (≥ 5.4 cm^3^), amniotic fluid levels (normal to oligohydramnios), gestational age at first evidence of oligohydramnios	Bladder volume (> 40 cc), renal echogenicity (normal to hyperechogenic), bladder refill (observed/not observed) between 18 and 30 weeks
Biochemical markers	Poorer prognosis if ≥ 3 abnormal markers among: Sodium < 100 mEq/L, Chloride < 90 mEq/L, Calcium < 8 mg/dL, Osmolality < 200 mOsm/L, β2‐microglobulin < 6 mg/L	None	Score 2 if ≥ 3 tested markers are abnormal among: Sodium < 100 mmol/L, Chloride < 90 mmol/L, Calcium < 8 mg/dL, Osmolality < 200 mOsm/L, β2‐microglobulin < 6 mg/L
Treatment guidance	Stage‐specific: Stage I (surveillance), Stage II (VAS or cystoscopy), Stage III (VAS may prevent pulmonary hypoplasia), Stage IV (no intervention or serial amnioinfusions as a bridge to postnatal dialysis and renal transplant)	Stage‐specific: Stage I (observation), Stage II (consider intervention), Stage III (high risk, observational)	Score‐based: Scores > 3 (poor prognosis, no intervention), Scores 0–3 (potential benefit from intervention)
Stages covered	I to IV	I to III	Not specified as stages, uses scoring system

## 4 Representative Clinical Illustrations

To complement the theoretical comparison of staging systems, Table 5 presents four representative, de‐identified clinical cases, each corresponding to one of the reviewed staging systems. These examples include fetuses diagnosed with LUTO at a tertiary referral center and either managed expectantly or treated with prenatal intervention. Each case includes prenatal findings, staging classification, the clinical decision made, and the postnatal outcome. Although these cases are not part of a formal validation analysis, they serve to illustrate how each classification system may guide decision making and predict clinical prognosis.

**TABLE 5:** Comparative clinical application of three prenatal LUTO staging systems in four representative cases.

Case/Figure	GA at diagnosis	Bladder volume	Kidney echogenicity	Cortical Cysts	AFI	Fetal biochemistry	Ruano stage	Fontanella stage	Nassr Score	Fetal therapy	Postnatal outcome
Case 1/Figure 2	20w0d	Enlarged	Normal	Absent	Normal	Favorable (Na^+^ 80 mmol/L, Cl^−^ 75 mmol/L, Osm 165 mOsm/L)	I	Mild	0	None	No pulmonary distress; normal renal function at 3 years
Case 2/Figure 3	19w0d	Severely enlarged	Normal	Absent	Anhydramnios	Favorable (Na^+^ 85 mmol/L, Cl^−^ 80 mmol/L, Osm 175 mOsm/L)	II	Severe	0	Fetal cystoscopy + VAS (×2)	No pulmonary distress; normal renal function with ongoing vesicostomy
Case 3/Figure 4	19w0d	Severely enlarged	Mild hyperechogenicity	Absent	Severe oligohydramnios	Not favorable (Na^+^ 105 mmol/L, Cl^−^ 95 mmol/L, Osm 205 mOsm/L)	III	Severe	3	Fetal vesicoamniotic shunt	No pulmonary distress; mild renal dysfunction at 1 year
Case 4/Figure 5	20w0d	Mildly enlarged	Bilateral hyperechogenic with dysplasia	Present	Anhydramnios	Not performed	IV	Severe	8	None	Severe pulmonary hypoplasia; neonatal death within 6 h

Abbreviations: AFI, Amniotic Fluid Index; Cl−, Chloride; GA, Gestational Age; Na+, Sodium; Osm, Osmolality; VAS, Vesicoamniotic Shunt.

### 4.1 Case 1

**FIGURE 2:**
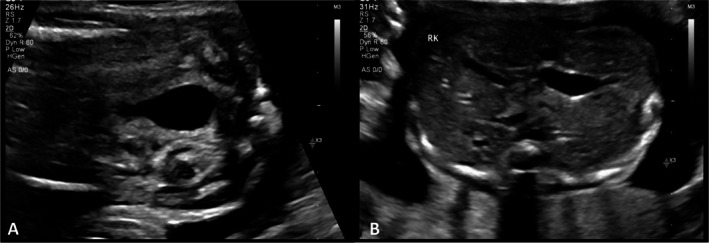
Prenatal ultrasound findings in Case 1. (A) Enlarged fetal bladder with thickened wall and “keyhole sign” at 20 weeks' gestation. (B) Bilateral hydronephrosis with normal renal echogenicity and no cortical cysts.

A pregnant woman, gravida 1 para 0, was referred to our Fetal Center at 19 weeks' gestation due to an enlarged fetal bladder with dilated posterior urethra (“keyhole sign”) and thick bladder wall (Figure 2A), in association with bilateral hydronephrosis but without cortical cysts and with normal echogenic kidneys (Figure 2B). Amniotic fluid volume was normal, and both fetal karyotype and microarray were normal. Fetal urinary biochemistry revealed sodium 80 mEq/L, chloride 75 mEq/L, and osmolality 165 mOsm/L. Bladder refill was complete (100%) 48 h after ultrasound‐guided vesicocentesis. These findings were consistent with Ruano Stage I, reflecting mild obstruction with preserved renal and bladder function. The patient was followed conservatively with weekly ultrasound surveillance. At 39 weeks, she delivered a 3500 g male infant with Apgar scores of 9, 10, and 10. Postnatal evaluation confirmed a small partial posterior urethral valve, treated successfully with cystoscopic ablation. The infant had an uneventful neonatal course and remains well with normal renal function at 3 years of age. According to the Fontanella system, this fetus would be classified as Mild LUTO, given the absence of oligohydramnios at 26 weeks. The Nassr score was calculated as 0, with no abnormal parameters. All three systems agreed on the benign nature of this case, although only Ruano's framework explicitly integrates biochemistry and functional refill into the classification, offering a structured rationale for conservative management.

### 4.2 Case 2

**FIGURE 3:**
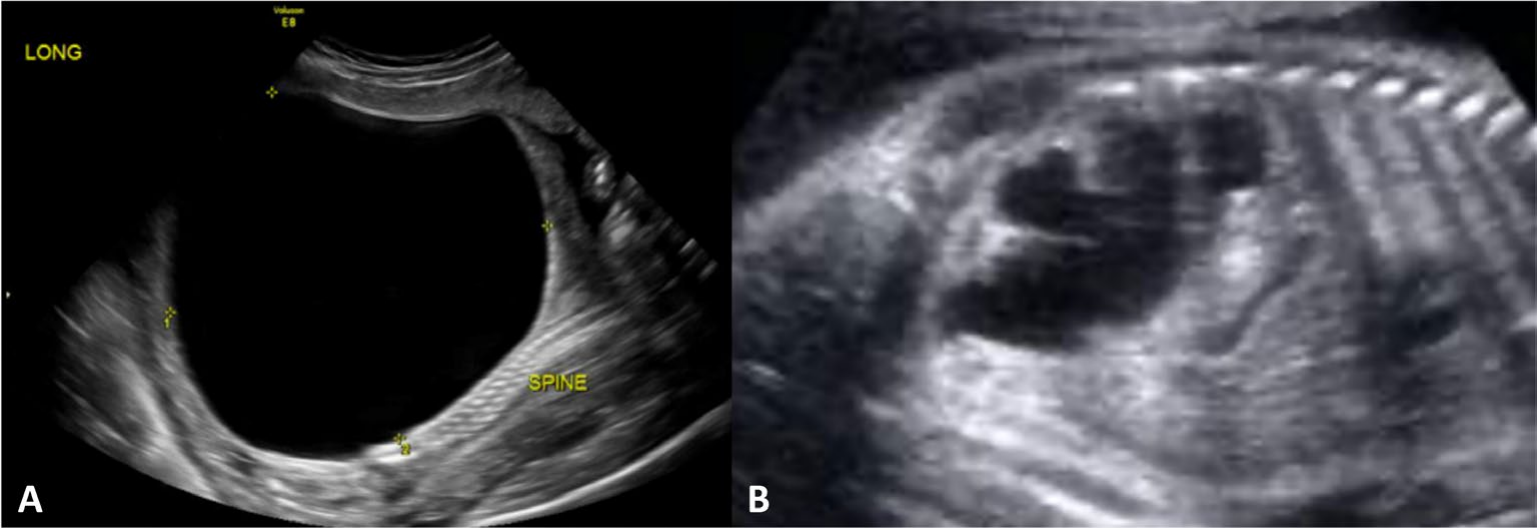
Prenatal ultrasound findings in Case 2. (A) Severely distended fetal bladder with thickened wall and “keyhole sign” at 19 weeks' gestation. (B) Bilateral hydronephrosis with normal renal echogenicity and absence of cortical cysts.

A gravida 2 para 1 patient was referred at 19 weeks' gestation for evaluation of an extremely distended fetal bladder with a thick wall and “keyhole sign” (Figure 3A), associated with bilateral hydronephrosis and normal echogenic kidneys (Figure 3B). Anhydramnios was noted. Fetal urine biochemistry demonstrated sodium 85 mEq/L, chloride 80 mEq/L, and osmolality 175 mOsm/L. Bladder refill was 90% after vesicocentesis. These findings were consistent with Ruano Stage II, characterized by significant obstruction with preserved renal potential, and fetal intervention was recommended. Fetal cystoscopy was performed and suggested urethral atresia. A vesicoamniotic shunt was placed, and a second shunt was later inserted at 28 weeks due to migration. Follow‐up ultrasounds showed normal amniotic fluid volume and decompressed bladder. At 33 weeks, the patient delivered a 2900 g male infant with Apgar scores of 8, 9, and 9. Postnatal diagnosis confirmed urethral atresia, requiring vesicostomy. At 4 years, the child maintains normal renal and respiratory function, although vesicostomy is still in place. Retrospectively, Fontanella's system would classify this as Severe LUTO, due to the presence of anhydramnios before 20 weeks, though the system does not offer intervention thresholds. The Nassr score was 0, based on favorable biochemistry, refill, and normal morphology, which supports intervention. This case illustrates how Ruano and Nassr better capture the positive renal potential and justify active management, while Fontanella lacks the granularity to differentiate favorable from high‐risk phenotypes.

### 4.3 Case 3

**FIGURE 4:**
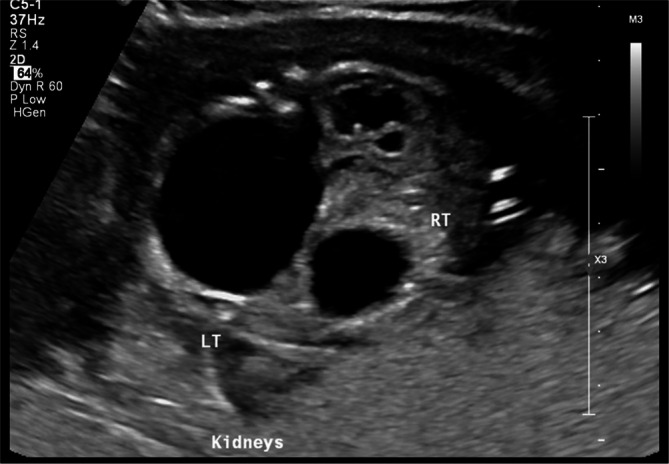
Prenatal ultrasound image in Case 3 at 19 weeks' gestation. Severely enlarged fetal bladder and bilateral hydronephrosis with mildly hyperechogenic kidneys and preserved corticomedullary differentiation. No cortical cysts are visible.

A 19‐week fetus in a primigravida presented with a severely enlarged bladder, bilateral hydronephrosis, and mild hyperechogenicity of the kidneys (Figure 4). Cortical cysts were absent. Amniotic fluid was severely reduced. Fetal urine biochemistry showed sodium 105 mEq/L, chloride 95 mEq/L, and osmolality 205 mOsm/L—values suggestive of compromised renal function. Bladder refill following vesicocentesis was incomplete (50%). Based on these findings, the case was classified as Ruano Stage III, representing a more advanced obstruction with unfavorable biochemistry and incomplete refill. A vesicoamniotic shunt was placed, and follow‐up scans demonstrated normalized amniotic fluid and decompressed bladder. Delivery occurred at 34 weeks. The 3250 g male neonate showed no respiratory distress and underwent postnatal cystoscopic fulguration of posterior urethral valves. He was discharged after 2 months with elevated but non‐dialysis‐requiring creatinine. At 1 year, the infant remains with mild renal impairment. Fontanella's system would again classify this as Severe LUTO but does not account for the unfavorable biochemistry and refill parameters. The Nassr score was 3, placing the case at the threshold of benefit for intervention. While Nassr and Ruano systems both captured the risk, only Ruano provides a stage‐specific rationale with therapeutic direction. Fontanella's classification lacks sensitivity to functional data and does not differentiate between salvageable and progressive disease.

### 4.4 Case 4

**FIGURE 5:**
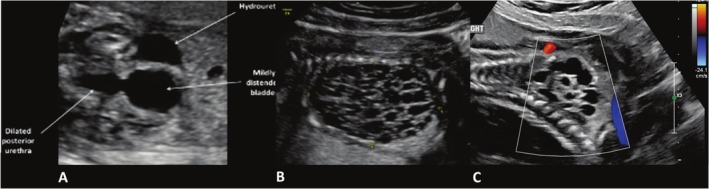
Prenatal ultrasound findings in Case 4. (A) Mildly distended fetal bladder and dilated posterior urethra (“keyhole sign”) at 20 weeks' gestation. (B) Bilateral hyperechogenic kidneys with multiple cortical cysts consistent with renal dysplasia. (C) Color Doppler confirming severe parenchymal damage with absent corticomedullary differentiation.

A primigravida was referred at 20 weeks for evaluation of a mildly enlarged fetal bladder and thickened wall with “keyhole sign” (Figure 5A), associated with anhydramnios and bilateral cystic renal dysplasia (Figure 5B–C). No fetal biochemistry was performed due to absent fluid. A diagnostic amnioinfusion confirmed renal non‐function. The findings were diagnostic of Ruano Stage IV, which is characterized by anuria, anhydramnios, and established renal failure. Weekly ultrasound monitoring was continued, and labor was induced at 39 weeks. A 3600 g male infant was delivered vaginally and died within 6 h due to severe pulmonary hypoplasia and renal failure (creatinine 3.6 mg/dL). In this case, Fontanella again classifies the presentation as Severe LUTO, but does not provide exclusionary criteria for non‐intervention. The Nassr score was retrospectively estimated at 8—the maximum possible—due to multiple risk factors. Both Ruano and Nassr clearly define this as a non‐interventional scenario, whereas Fontanella fails to distinguish cases of irreversible renal failure from treatable severe obstruction. This case underscores the value of a structured staging system that incorporates biochemistry and functional markers for accurate prognostication.

## 5 Feasibility and Implementation Barriers

The practical implementation of prenatal LUTO staging systems is closely tied to institutional resources, technical expertise, and access to specialized diagnostic modalities. The (Ruano et al. 2017) and Nassr (Nassr et al. 2021) systems both rely on fetal urine sampling obtained via vesicocentesis, which requires specialized procedural skills, access to high‐resolution ultrasound, and prompt laboratory processing of fetal urinary biochemical parameters, including sodium, chloride, calcium, osmolality, and β2‐microglobulin. However, in selected cases of anhydramnios, a preliminary amnioinfusion may be performed to transiently re‐establish a measurable fluid pocket, thereby enabling safe amniocentesis for diagnostic purposes (O'Hare et al. 2019). This approach is not routinely feasible in all fetal medicine units, particularly in resource‐limited settings, but may be considered in specialized centers with appropriate expertise and strict procedural safeguards. By contrast, the (Fontanella et al. 2019) system, based solely on sonographic parameters such as bladder volume and amniotic fluid index, can be implemented using standard prenatal imaging protocols. However, its lack of biochemical correlation may limit its sensitivity in detecting early renal compromise, thereby reducing its utility in nuanced decision‐making regarding fetal intervention.

## 6 Conclusion

The three proposed prenatal staging systems for fetal LUTO have similarities and particularities. The Ruano's prenatal staging system seems to offer a more comprehensive stratification of different severities of fetal LUTO as well as guidance for potential prenatal therapy based on the 4 stages. Nevertheless, no prenatal staging system for LUTO has been validated in large‐scale, multicenter clinical studies. Future research should focus on prospective multicenter studies designed to compare the predictive value of existing prenatal staging systems using standardized imaging protocols and blinded assessment of postnatal outcomes. Such efforts will be essential to establish reliable, evidence‐based prognostic tools for fetuses with LUTO and optimize patient selection for fetal intervention.

## Author Contributions

Conceptualization by U.M.P., G.P., and R.R.; methodology, validation, and resources by U.M.P, G.P., and R.R.; investigation by U.M.P, I.P., M.P., G.P., and R.R.; data curation, writing – original draft preparation, visualization and project administration by I.P., M.P., U.M.P.; writing – review and editing by C.P.K., A.A., M.D.K., G.P. and R.R. All authors have read and agreed to the published version of the manuscript.

## Ethics Statement

The authors have nothing to report.

## Consent

The authors have nothing to report.

## Conflicts of Interest

The authors declare no conflicts of interest.

## Data Availability

The data that support the findings of this study are available on request from the corresponding author. The data are not publicly available due to privacy or ethical restrictions.
